# Evaluation of Clinical and Histological Outcomes of Adipose-Derived Mesenchymal Stem Cells in a Rabbit Corneal Alkali Burn Model

**DOI:** 10.1155/2021/6610023

**Published:** 2021-03-07

**Authors:** Diamantis Almaliotis, Angelos Thomas, Anastasia Komnenou, Eleni Gounari, Stavroula Almpanidou, Thomas Siempis, Nikolaos Papaioannou, Georgios Koliakos, Eleni Papakonstantinou, Konstadinos Sotiropulos, Vasileios Karampatakis

**Affiliations:** ^1^Laboratory of Experimental Ophthalmology, School of Medicine, Aristotle University of Thessaloniki, Thessaloniki, Greece; ^2^School of Veterinary Medicine, Faculty of Health Sciences, Aristotle University of Thessaloniki, Thessaloniki, Greece; ^3^Department of Biological Chemistry, School of Medicine, Aristotle University of Thessaloniki, Thessaloniki, Greece; ^4^Biohellenika, Biotechnology Company, 57001 Thessaloniki, Greece; ^5^Department of Pharmacology, School of Medicine, Aristotle University of Thessaloniki, Thessaloniki, Greece; ^6^Army Share Fund Hospital, Athens, Greece

## Abstract

To assess effects of adipose-derived mesenchymal stem cells (AMSCs) in corneal alkali injuries in an experimental animal model. Twenty white New Zealand rabbits were included in the study. The animal models were randomly divided into 2 groups. Rabbits in the AMSC group (*n* = 10) received an intrastromal, a subconjunctival injection, and topical instillation of 0.5 ml totally of phosphate-buffered saline (PBS) containing 2 × 10^6^ AMSCs. In the control group (*n* = 10), rabbits received only 0.5 ml of PBS using the same methods. A masked investigator measured the corneal sensation, anterior chamber Inflammation (ACI), and conjunctival congestion. Additionally, a blind histological and immunohistochemical evaluation was made. In the AMSC group, the central corneal sensation was increased whereas ACI and conjunctival congestion were reduced compared to the control group in the 28 days of follow-up (*p* < 0.05). A statistically significant difference (*p* < 0.05) was noted between the two groups as recorded in the above parameters. Histological analysis showed that pathological vascularization was markedly reduced in the AMSC group which was consistent with the absence of factor VIII in the immunohistochemistry sections. There is a trend towards improved clinical outcomes including corneal sensation as well as acceleration in the restoration of normal corneal architecture in corneal alkali burns treated with AMSCs, results that support the need for further research in the field.

## 1. Introduction

Alkali burns of the cornea are a common ophthalmologic emergency, accounting for 60% of ocular burns [1]. Alkali agents are lipophilic, penetrating tissues more rapidly than acids and can therefore produce extensive damage to the ocular surface through four phases, including immediate, acute, early, and late repair phases, leading potentially to blindness [2].

The initial clinical management during the acute phase with copious irrigation of the affected eye is critical in limiting the duration of chemical exposure [3, 4]. The administration of anti-inflammatory and antiangiogenic agents as well as of substances enhancing epithelial healing is also recommended [3, 4]. However, a more definitive surgical management including limbal stem cell transplantation or amniotic membrane grafting is usually needed [5, 6]. Corneal transplantation has the limitation of the possibility of graft rejection and in cases of extensive limbal stem cell deficiency, which we often see in chemical injuries, it is insufficient to manage these injuries [7].

Chemical injuries are also known as a cause of impairment of the corneal sensation [8]. The corneal sensation is an essential outcome because corneal nerves are important for maintaining limbal stem cell homeostasis and function. A neurotrophic cornea undergoing corneal transplantation has a high risk for complications such as persistent epithelial defects, corneal ulceration, melting, and sometimes perforation [8]. Anterior chamber inflammatory reaction is more common in alkaline injuries because of the greater depth of penetration. The eyes with alkaline injuries may be also sometimes complicated by the deterioration of the tear production due to the damage of lacrimal, conjunctival, and eyelid glands, with consequent corneal dryness, abrasions, ulcers, and perforations [9].

In facing such difficulties, a novel therapeutic model has been experimentally evaluated with favorable clinical and immunohistochemical outcomes [10]. This method uses adipose-derived mesenchymal stem cells (AMSCs) injected intrastromally and subconjunctivally as well as applied topically [10]. The rationale behind the therapeutic use of AMSCs is that they possess attractive properties such as improved tissue repair or even regeneration, immunomodulatory abilities, and seem to be well-tolerated when used in allogeneic transplantation which makes them suitable in cell-based tissue engineering [10, 11].

AMSCs are undifferentiated cells that can multiply, regenerate, and transform into differentiated cells [12]. The bone marrow is a major source of tissue-derived AMSCs [13]. They can also be isolated from adipose or oral tissue, umbilical cord blood, bone marrow, etc. [14–16]. There has been a recent surge in studies evaluating the use of AMSCs in ocular surface disease including cases of corneal injury [17–20].

In the present study, we investigated the therapeutic effect of the treatment method in some additional but clinically important outcomes such as corneal sensation, anterior chamber inflammation (ACI), and conjunctival congestion (CC), which to our knowledge have not been described in other studies (except for the conjunctival congestion) [10].

At the same time, we present the associated histological changes in both treatment and control groups along with an immunohistochemical analysis for the presence of factor VIII which is a marker for neovascularization [21]. All these results were not evaluated in our previously published article, and we assume that they represent an important addition to the available literature.

## 2. Materials and Methods

### 2.1. Animals

All procedures in this study were performed and approved by the local Ethics Committee of the Aristotle University of Thessaloniki, as well as by the Committee of the Department of Veterinary Medicine of Thessaloniki (436002/3803) and conformed to the ARVO Statement for the Use of Animals in Ophthalmic and Vision Research and the European Communities Council Directive (86/609/EEC) [22]. The study was conducted in the Companion Animal Clinic, Faculty of Veterinary Medicine, Aristotle University of Thessaloniki (EL54BIO16). Before the study, a complete and thorough ophthalmic examination was performed in all rabbits to ensure that they were free of any ocular pathologic conditions.

Twenty New Zealand rabbits 6 months old (10 males/10 females) weighing 3.5-4.2 kg were anesthetized using intramuscular xylazine (3–5 mg/kg) and ketamine (30–50 mg/kg) as well as topical eye drops of proparacaine hydrochloride 0.5% mixed with Povidone Iodine 5%.

### 2.2. Isolation and Culture of AMSCs

The AMSCs were obtained from the inguinal fat tissue of rabbits and were prepared for administration. Specifically, the AMSCs isolated from the rabbits were washed with PBS and then further processed with 0.5 mg/ml collagenase type-1 (Sigma, Aldrich, MO, St. Louis) for 1 h at 37°C whilst they were constantly shaken. After the addition of an equal amount of PBS and incubation of this solution, three distinct layers were formed. The AMSC layer (middle) was aspirated after 10 minutes of centrifugation at 600 × g, and the cellular pellet created was suspended in a special medium (Dulbecco's modified Eagle's medium) supplemented with 5% fetal calf serum (Atlanta Biologicals Atlanta, Georgia) and penicillin (100 IU/ml) (Sigma Inc., St. Louis, MO, USA), and streptomycin (100 *μ*g/ml) (Sigma Inc., St. Louis, MO, USA) was also added. Finally, the above solution was cultured in a humidified chamber (5% CO2) at 37° with medium changes every 2-3 days. AMSCs were characterized for their main mesenchymal characteristics and administered to rabbits between passages 3-4.

## 3. Characterization of AMSC with Flow Cytometry and Induced Differentiation

Regarding the characterization of AMSCs, flow cytometry was performed for the detection of typical surface marker expression. Briefly, upon detachment of cells with Trypsin-EDTA 1x in PBS and mild centrifugation, staining with monoclonal antibodies (mAbs) CD73, CD105, CD90, and CD44 was performed for 15 min in absence of light. Results were obtained on a FACSCalibur device (Becton Dickinson, BD, Franklin Lakes, NJ, USA) [23] and analyzed with the CellQuest Pro6 software.

To test AMSC differentiation capacity, they were seeded onto 24-well plates at a cellular concentration of 2∗10^4^ per well. *Τ*heir osteogenic, adipogenic, and chondrogenic ability was evaluated after the addition of appropriate mediums in MSC cultures for 28-32 days with medium changes every 2-3 days and incubation in 37°C with 5% CO_2_. After the end of induced differentiation, stainings (Alizarin Red, Oil Red, Alcian blue) were performed related to each differentiation.

### 3.1. Animal Model of Corneal Alkali Burn

A corneal alkali burn was generated in the right eye of each rabbit (20 rabbits) using a 6 mm diameter Whatman Filter Paper soaked in 1 N NaOH. This was applied to the cornea for 30 s, 1/2 of the disc covered of the upper peripheral cornea and the other half the upper bulbar c*ο*njunctiva, followed by rinsing with the balanced salt solution for 1 minute.

### 3.2. Initial Medical Management

The animals received the following combination of drops as part of the initial medical management, which is usually be applied in respective injuries in humans [24]. Drops of dexamethasone 0.1% and tobramycin 0.1% (Tobradex, Alcon Fort Worth, Texas) were instilled four times daily for the first week along with a mydriatic (Cyclopentolate 0.5% by Alcon) once a day for 10 days.During the second week, drops of tobramycin 0.1% (Tobrex, Alcon Fort Worth, Texas), as well as drops of nepafenac 0.1% were instilled four times daily (Nevanac, Alcon Fort Worth, Texas). Citrate 10% and ascorbate 10% were also applied topically four times daily for the first 2 weeks. Ascorbic acid is known to improve corneal wound healing by promoting the synthesis of collagen by corneal fibroblasts [24]. Citric acid inhibits neutrophil activity thus reducing the inflammatory response [24].

### 3.3. Topical, Intrastromal, and Subconjunctival Injection of AMSCs

After the induction of the alkali burn, the rabbits were divided randomly into 2 groups. In the first group (*n* = 10), the rabbits received 0.5 ml PBS containing 2 × 10^6^ AMSCs. The application was performed in 3 ways: a subconjunctival injection (with an insulin syringe gauge) and a second injection in the corneal stroma (in the healthy tissue near the wound site through pockets created by 15° knife) as well as a topical application of the same solution to the cornea in the form of drops. In the control group (*n* = 10), the same protocol was followed, but the PBS did not contain any AMSCs. All rabbits in each group were clinically evaluated as described below.

### 3.4. Observation and Examination

To minimize the interaction between the various measurement for the ophthalmological evaluation, minimally invasive techniques were performed. On this basis, the examinations were performed according to the following sequence: biomicroscopy of the conjunctiva, cornea, and anterior chamber; photography for the assessment of outcomes; corneal aesthesiometry; euthanasia; histological sections; and immunohistochemical analysis. Each rabbit underwent a thorough examination, in the follow-up period starting on the day of the alkali burn as well as at postoperative 3, 7, 14, 21, and 28 days. The process of euthanasia was done after the 28th day. Clinical evaluation was performed by a blinded ophthalmologist who was not aware of the group to which the rabbit had been allocated. This was done with a portable slit lamp (Kowaportable, SL-15®, Kowa, Tokyo, Japan) and included the grading of the conjunctival congestion and ACI. Each parameter was graded between 0 and 3 as described in the following table (Table 1).

### 3.5. Aesthesiometry

Aesthesiometry was performed with the Cochet-Bonet special corneal aesthesiometer which measures the threshold of a corneal touch (Corneal Touch Threshold (CTT)). The Cochet-Bonnet aesthesiometer is applied to the corneal surface using nylon filaments of variable diameter and length. The thread of the aesthesiometer should be perpendicular to the cornea. Its maximum length should be 6 cm, and the length is gradually reduced by 0.5 cm. A shorter length indicates decreased sensation [25]. The normal values of corneal aesthesiometry in normal rabbits are 2.63 ± 0.63 cm [26]. The test was performed on the burn site as well as in the central section of the cornea and was repeated 5 times with each thread length. Besides, we also checked the length for which there was a reaction after 3-5 consecutive corneal stimuli in the corneal alkali burn site. This was recorded in cm and was considered as in the threshold of CTT.

### 3.6. Histological Examination

The samples were evaluated by a masked examination. Corneal sections were taken from the burn site of every rabbit and were obtained and embedded in 10% formalin and paraffin (Paraplast Plus, Tissue Embedding Medium, Leica). The sections were 2.5 *μ*m thick and were stained with hematoxylin and eosin to evaluate the connective tissue (Bio-Optica, Milan, Italy). All samples were evaluated for healing, the presence of inflammation, and neovascularization.

### 3.7. Immunohistochemistry

Immunohistochemistry was performed on corneal sections which had been embedded with paraffin (2 *μ*m). Factor VIII is an antigen (a marker of pathological angiogenesis) (FVIII, 1 : 300 Abcam), which was visualized by Dako Real Envision Detection System peroxidase/DAB +. Following that, the samples were counterstained with hematoxylin to be studied by light microscopy.

### 3.8. Statistical Analysis

Statistical analysis was done with the SPSS package (SPSS®, Chicago, IL), version 22. Parametric data distribution was checked with Shapiro-Wilk analysis. Nonparametric data were analyzed with the Mann–Whitney *U* method between the study groups. *p* values less than 0.05 were considered statistically significant.

## 4. Results

### 4.1. AMSC Characterization

**Figure 1:**
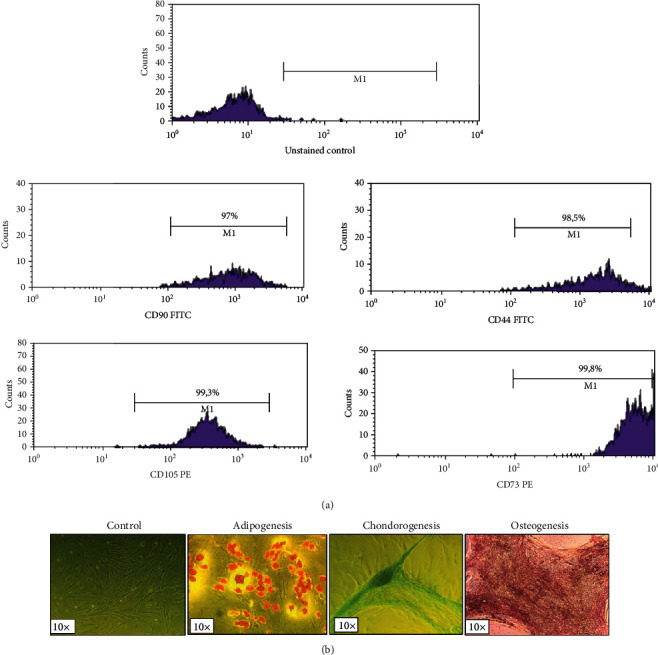
Characterization of administered AMSCs (a) with flow cytometry and (b) with specific stainings showing their differentiation capacity.

Flow cytometric results regarding surface marker expression revealed that AMSCs were highly positive for CD90, CD105, CD73, and CD44 typical surface markers (Figure 1(a)). The induced differentiation of AMSCs revealed their osteogenic, adipogenic, and chondrogenic capacity as confirmed with positive alizarin, Oil Red, and Alcian stainings, respectively, and in comparison with the control unstained groups (Figures 1(a) and 1(b)).

### 4.2. Corneal Aesthesiometry

In the AMSC group, the mean value of the threshold was within normal limits and compared to the control group at all time points. These values were recorded from day 3 until the last day of follow-up (day 28). A statistically significant difference (*p* < 0.05) was noted between the two groups, regarding the central cornea on the 7th and the 21th day (see Table 2(a)). Concerning the sensitivity in the burn site, a statistically significant difference (*p* < 0.05) was also noted between the two groups on the 21th and 28th day (see Table 2(b)).

### 4.3. Conjunctival Congestion

**Figure 2:**
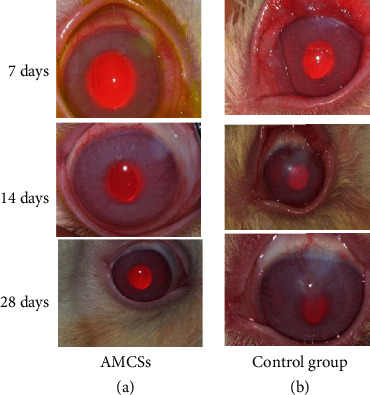
Representative photographs demonstrating the conjunctival congestion of rabbits from AMSCs and the control group on days 7, 14, and 28 after alkali burn.

In both groups, the mean value of conjunctival congestion reached its highest level on the 3rd day after the alkali burn. Over the next few days, conjunctival congestion started to decrease in both groups; however, the reduction was greater in the AMSC group (Table 3; statistically significant differences (*p* < 0.05) were noted between the two groups, in the 7th and 14th day). The change in the mean value of conjunctival congestion followed the same decreasing course in both groups until the 28th day, and this is represented in Figure 2.

### 4.4. Anterior Chamber Inflammation

**Figure 3:**
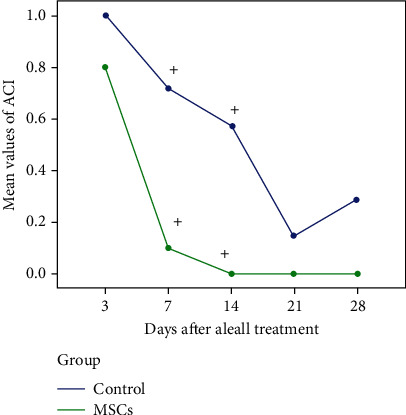
The pattern of change of the mean value of ACI (statistically significant difference (*p* < 0.05), included in figure with black asterisks). Statistically significant differences were noted between AMSCs and the control group on the 7th and 14th day.

The mean values of anterior chamber inflammation on the 3rd day after alkali burns increased for both groups. These values started to decrease in both groups in the following days. The pattern of change of the mean value of the ACI is similar for both groups and followed a decreasing pattern. Nevertheless, the reduction in ACI was more rapid in the AMSC group, and it is worth noting that on the 14th day, there was no residual ACI. In Table 3, statistically significant differences were noted between AMSCs and the control group on the 7th and 14th day (Figure 3). The ACI in the control group appeared to increase again on the 28th day.

### 4.5. Histological Examination

**Figure 4:**
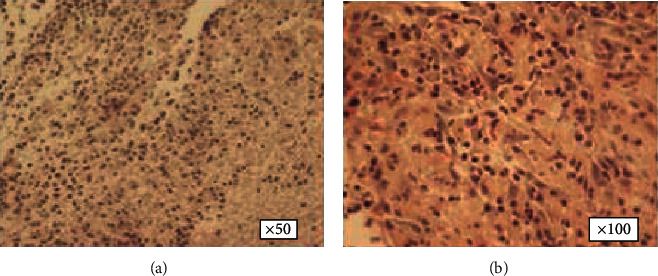
(a, b) Normal corneal tissue sections of rabbits.

The results of the histological examination of both groups are presented in the following figures.  Figure 4 depicts normal corneal tissue.

**Figure 5:**
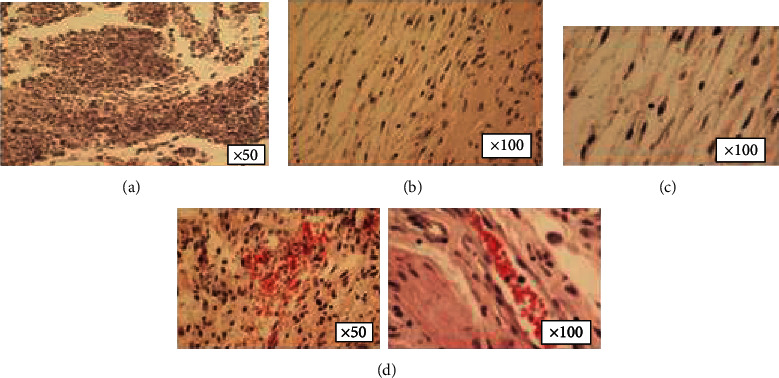
(a–d) In the control group, the tissues of the cornea lost their normal architecture and appeared to have an irregular alignment of their collagen fibers. There was also increased neovascularization.

In the control group, the corneal tissues lost their normal architecture and appeared to have an irregular alignment of their collagen fibers. There was also increased pathological vascularization, characterized by multiple red blood cells which are visible in the transverse cuts of the vessels (Figures 5(a)–5(d)).

**Figure 6:**
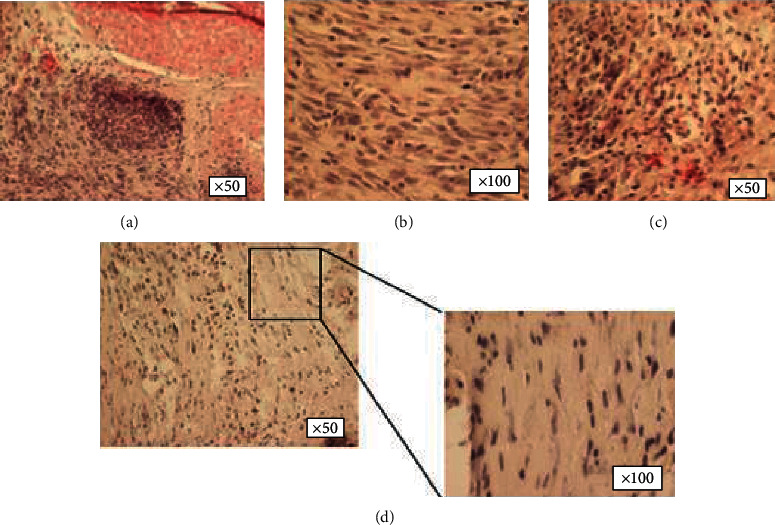
(a–d) The pathological vascularization in the AMSC group is markedly reduced compared to the control group. Cells with elongated nuclei could correspond to AMSCs which randomly migrated to the tissue for targeted repair.

In the AMSC group, there was an increased inflammatory reaction with the accumulation of macrophages in the area of regenerative activity (Figure 6). The pathological vascularization in the AMSC group is markedly reduced compared to the control group. It should be pointed out that the appearance of cells with elongated nuclei, in multiple sites without uniform distribution in a single cell layer, probably indicates the migration of AMSCs in the tissue, which exert their therapeutic action (Figures 6(c) and 6(d)).

### 4.6. Factor VIII

**Figure 7:**
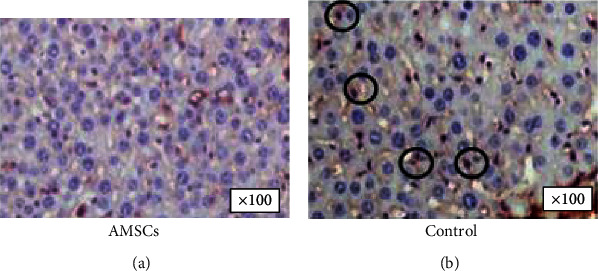
(a, b) Histological sections of factor VIII in the electronic microscope in the AMSC group (a) and control group, respectively (b). The presence of factor VIII is highlighted by the circled areas in (b).

10 samples were examined in the anterior stroma. In the control eyes, *ο*ur results show that factor VIII was not present in the AMSC group. The presence of vessels and fibrous septae in the corneal sections was in the whole control group that confirmed the pathological neovascularization, which was not present in any sample in the AMSC group (Figures 7(a) and 7(b)).

## 5. Discussion

The therapeutic effects of AMSCs are thought to be based on their anti-inflammatory action, inhibition of corneal angiogenesis along with the ability to differentiate into various cell types including corneal epithelium, all of which have been proven in various studies [10, 12, 27, 28]. The exact mechanism of action of AMSCs is not yet fully understood, but it is thought to be secondary to the secretion of growth factors to facilitate the survival of injured cells and the promotion of tissue regeneration in the microenvironment [29, 30].

The improved clinical and immunohistochemical outcomes associated with the use AMSC cells derived from inguinal adipose tissue of rabbits and administered by subconjunctival and intrastromal injection as well as a topical application in the acute stage of the corneal alkali injury have been described in our previous paper [10]. In that study, we demonstrated that they reduce the associated corneal neovascularization and residual corneal opacity along with promoting more prompt healing of the injured cornea.

What this study adds is the confirmation that chemical burns cause an impaired corneal sensation at the burn site especially and at the central cornea as measured with the Cochet-Bonet corneal aesthesiometer, and there was a trend of normalization in the corneal sensation of the AMSC treated group. To our knowledge, corneal aesthesiometry measurements using the Cochet-Bonet aesthesiometer have not been reported in the literature in cases of chemical burns. It is an important factor in the overall clinical assessment of a patient with a chemical burn, and it should be assessed when the effect of the anesthetic drops (after the initial irrigation) has worn off [31]. In our opinion, the favorable results in terms of the improvement in the central corneal sensation noted in the treatment group are promising as they would be beneficial concerning the outcome of subsequent corneal surgery in the form of a keratoplasty. Along with limbal stem cell deficiency and corneal vascularization, reduced sensation in an ocular surface is associated with a high risk of failure of corneal keratoplasty [32–34]. The role of neurons in corneal health, healing, scarring, and immunology is of great importance. Therefore, in primary sensory neurons, the molecular and cellular changes that occur as a disease or trauma effects, including their regenerative processes, are a high priority. Several ocular pathologies affecting the cornea such as chemical burns, diabetes, and neurotrophic keratitis are related to the impairment of corneal innervation that further has a detrimental impact on wound healing [35]. It is well known that adipose-derived MSCs exhibit a wide range of anti-inflammatory and immunomodulatory properties [36]. Several studies have shown that adipose-derived MSCs provide growth factors, neuromediators, and nutrients to the ocular surface that promote the healing process and corneal nerve regeneration [37]. Furthermore, MSCs through their paracrine activity promote survival and endogenous repair of injured retinal ganglion cells in glaucomatous eyes since they produce large amounts of neurotrophic factors [35]. In our study, it was shown the positive effect of MSCs on restoring corneal nerve function and sensitivity.

As concerns ACI, it was reduced in the AMSC group both on day 3 and day 7 after the alkali burn and was not present after day 10 whereas in the control group, there was residual ACI until the 28th day. Statistically significant differences (*p* < 0.05) were noted between the two groups, in the 7th and 14th day. Concerning the observed conjunctival congestion, this was similarly reduced in the AMSC group on days 3 and 7, and after that, the conjunctiva was classified as normal in terms of the presence of hyperemia. In the control group, there was residual conjunctival congestion throughout the entire observation period (28 days). Statistically significant differences (*p* < 0.05) were noted between the two groups, in the 7th and 14th day. The aforementioned observations are indicative that the AMSC treated eyes are less inflamed from early on in the immediate treatment period, and this is continued until the end of the follow-up (28 days). Thus, AMSC treatment may make tissues more willing to surgeries. A reaction is a sign of ongoing inflammation in the eye, and the eyes with inflammation are at higher risk of rejection if corneal transplantation is to be considered [38, 39], and any intraocular inflammation should be treated before the keratoplasty [38]. On that basis, the use of AMSCs in corneal alkali injuries as described above could contribute positively to the survival of potential in later stage corneal keratoplasty in the context of reduced corneal vascularization, improved corneal sensation, and reduced AC inflammation.

In our histological analysis, we demonstrated that the neovascularization of the cornea was reduced, and AMSCs were incorporated in the healing cornea which can explain their therapeutic effects (Figure 5(d)). Upon review of the relative published literature, there are studies with the use of AMSCs in chemical injuries that have produced results similar to our histological findings. The exact mechanism of corneal neovascularization has been a topic of wide discussion. Various components are known that can induce neovascularization of the corneas, including corneal injuries and wound repair [40, 41]. Additionally, the morphology of corneal new vessels has been widely studied and described by various techniques [42, 43].

Our present results are in agreement with all these previous observations and target to add new evidence on the morphological development of corneal neovascularization of different studies showing that the application of MSCs on the cornea can restrain neovascularization after chemical injuries [44, 45]. In our study, the formation of new vessels in the cornea was constricted during the process of wound healing in the AMSC group as compared to the controls, thereby showing an inhibitory impact of AMSCs on corneal neovascularization. Different routes of administration have been used for AMSC applications, such as intrastromal [46], subconjunctival [47], with nanofiber scaffolds [48], and systemically [49]. In our model, AMSC applied by three different routes effectively inhibited the formation of new vessels when administered immediately after the alkali burn.

Ke et al. studied the effect of subconjunctivally injected BM MSCs with and without topical application of a polysaccharide hydrogel in corneal alkali burns in rats [50]. Their histological analysis showed marked inflammation, neovascularization, and loss of architecture in the control group. On the contrary, both with and without polysaccharide hydrogel MSC groups showed more regularly aligned epithelial cells and reduced inflammation and neovascularization. The group treated with a combination of polysaccharide gel and MSCs showed the best improvement in histological examinations. In this particular study, the clinical outcomes in the groups that received MSCs either with or without polysaccharide were improved as well (increased corneal epithelization, reduced corneal opacity, and neovascularization). Lin et al. performed histological analysis of corneas with alkali injury not treated with MSCs and treated with a topical solution of mesenchymal stem cells derived from orbital fat tissue. The use of MSCs in their study promoted corneal reepithelialization, decreased stromal infiltration, and reduced corneal thickness in the central cornea and limbal area [51]. Despite the different source and administration methods of the AMSCs in this study, the results are consistent with our results both in the present study and in our earlier published work [10].

Regarding factor VIII-related antigen, otherwise known as von Willebrand factor (vWf), it is produced by endothelial cells and megakaryocytes. Its function is to mediate platelet adhesion to the walls of injured vessels. The immunohistochemical detection of this factor has been used to quantify angiogenesis in animal models [21]. It is not found in normal corneal tissue. In our sample, we found that factor VIII was not present in the AMSC group, and this correlates with the reduced corneal vascularization demonstrated in our earlier paper [10]. The factor VIII immunostaining has been used previously in animals as a marker for the evaluation of neovascularization in tumors [52]. However, there are no similar references in the literature correlating FVIII corneal levels with the application of AMSCs in corneal alkali burns.

## 6. Conclusions

Without a doubt, the use of AMSCs is a promising therapy for corneal alkali injuries, and this is supported by our study and relative literature. Most of these studies were associated with better clinical results in experimental animal models for the eyes that had been treated with mesenchymal stem cells. Various stem cell sources have been used until now such as limbal ones, bone marrow-derived, orbital fat, or adipose-derived stem cells as well as different routes of administration that have been tested. It is encouraging to see that a human clinical trial took place aiming to evaluate the safety and clinical efficacy of bone marrow-derived MSCs versus the established therapy with limbal epithelial cells in cases of limbal stem cell deficiency [18].

On the other hand, the use of varied AMSC protocols has also been increasingly criticized over the insufficient reproducibility of experimental findings [53]. Details of different procedures and parameters, including the status of tissues for AMSC isolation, cell sorting, ex vivo expansion, purification, phenotyping, and administration of cells as well as follow-up examinations, need to be fully documented to translate the therapeutic potential into a reproducible clinical efficacy and outcomes [54, 55].

In summary, we conclude that adipose-derived AMSCs administered simultaneously topically, subconjunctivally, and intrastromal in alkali corneal burns in our animal model, show a trend of improving the central corneal sensation and reduce the associated ACI and CC as well as demonstrate favorable histological and immunochemical results. Given the sight-threatening nature of chemical injuries, more research should be done in humans, to establish stem cells as a valid option in the management of corneal alkali burns.

## Figures and Tables

**Table 1:** Grading of conjunctival congestion and ACI.

Grade	Description CC	Description ACI
Grade 0	Normal conjunctiva	No cells were seen in the AC
Grade 1	Mild conjunctival congestion	6-15 cells
Grade 2	Moderate conjunctival congestion	16-25 cells
Grade 3	Severe conjunctival congestion	>25 cells

**(a):** 

Center	Group					
	Control			Stem cells		
	*N*	Mean	Std. deviation	*N*	Mean	Std. deviation
3rd day	5	2.25	0.35	5	2.75	0.35
7th day	5	3.00	1.50	5	1.50	1.78
21th day	5	1.75	1.77	5	0.75	0.35
28th day	4	3.00	0.71	4	2.50	2.00

**(b):** 

Burn site	Group					
	Control			Stem cells		
	*N*	Mean	Std. deviation	*N*	Mean	Std. deviation
3rd day						
7th day	5	0.00		5	0.75	1.06
21th day	5	2.00	1.41	5	0.25	0.35
28th day	5	1.00	1.30	5	0.50	0.00

**Table 3:** Mean values of the conjunctival congestion and anterior chamber inflammation in each rabbit group.

	CC			ACI		
Days	Control	AMSCs	*p*	Control	AMSCs	*p*
3	1.36 (0.92)	0.85 (0.56)	0.15	1.00 (0.58)	0.80 (0.42)	0.43
7	0.73 (0.91)	0.08 (0.28)	0.03^∗^	0.71 (0.49)	0.10 (0.32)	0.01^∗^
14	0.55 (0.93)	0.00 (0.00)	0.02^∗^	0.57 (0.54)	0.00 (0.00)	0.01^∗^
21	0.36 (0.92)	00 (0.00)	0.12	0.14 (0.38)	0.00 (0.00)	0.23
28	0.27 (0.91)	00 (0.00)	0.28	0.29 (0.49)	0.00 (0.00)	0.08

## Data Availability

The data used to support the findings of this study are available from the corresponding author upon request.
